# A Review of Structure Construction of Silk Fibroin Biomaterials from Single Structures to Multi-Level Structures

**DOI:** 10.3390/ijms18030237

**Published:** 2017-03-03

**Authors:** Yu Qi, Hui Wang, Kai Wei, Ya Yang, Ru-Yue Zheng, Ick Soo Kim, Ke-Qin Zhang

**Affiliations:** 1 National Engineering Laboratory for Modern Silk, College of Textile and Clothing Engineering, Soochow University, Suzhou 215123, China; qjy1540945317@163.com (Y.Q.); weikai@suda.edu.cn (K.W.); ldxyyang@126.com (Y.Y.); ruyuezheng92@126.com (R.-Y.Z.); 2 Nano Fusion Technology Research Lab, Interdisciplinary Cluster for Cutting Edge Research (ICCER), Division of Frontier Fibers, Institute for Fiber Engineering (IFES), Shinshu University, Ueda, Nagano 386 8567, Japan; kim@shinshu-u.ac.jp

**Keywords:** silk fibroin, structure, biomaterials

## Abstract

The biological performance of artificial biomaterials is closely related to their structure characteristics. Cell adhesion, migration, proliferation, and differentiation are all strongly affected by the different scale structures of biomaterials. Silk fibroin (SF), extracted mainly from silkworms, has become a popular biomaterial due to its excellent biocompatibility, exceptional mechanical properties, tunable degradation, ease of processing, and sufficient supply. As a material with excellent processability, SF can be processed into various forms with different structures, including particulate, fiber, film, and three-dimensional (3D) porous scaffolds. This review discusses and summarizes the various constructions of SF-based materials, from single structures to multi-level structures, and their applications. In combination with single structures, new techniques for creating special multi-level structures of SF-based materials, such as micropatterning and 3D-printing, are also briefly addressed.

## 1. Introduction

Silks are commonly defined as protein polymers, which are present in the glands of arthropods such as silkworms, spiders, scorpions, mites, and bees, and then spun into fibers during their metamorphosis. The composition, structure, and properties of silks collected from different sources show variation [1,2]. In recent years, silk from *Bombyx mori* (silkworm) has been discussed extensively due to its biocompatibility, robust mechanical performance, tunable degradation, ease of processing, sufficient supply, and ease of acquisition from the mature sericulture industry [1,2,3,4]. Silkworm silk has been used in the traditional textile industry for more than 4000 years; it is admired for its soft, pearly luster and good mechanical properties [3,5]. Silk is composed of two major proteins: silk fibroin (SF) and sericin. The glue-like sericin protein wraps around fibroin; it is generally soluble and can be removed by a thermo-chemical treatment, also known as degumming [2,6]. Silk fibroin, a natural fibrous protein, is a more biocompatible biomaterial than some commonly used biological polymers such as collagen and poly(l-lactic acid) (PLA) [7]. The application of SF as a biomaterial began centuries ago, with its use as sutures for wound treatment [2,8]. Due to their excellent performance, SF-based biomaterials have been found suitable for a variety of applications, including drug delivery [9], vascular tissue regeneration [10], skin wound dressing [11], and bone tissue scaffolds [12].

Both synthetic and natural polymers have been widely used as biomaterials in tissue engineering. While synthetic polymers are more easily obtained through simple processing and modifications, natural polymers offer better biocompatibility [13]. Biomaterials for tissue engineering applications must incorporate the following properties: (1) excellent biocompatibility in vivo; (2) optimized physical properties, especially mechanical properties; (3) ability for construction of topographic and morphological cues; (4) degradability with safe by-products; and (5) diffusivity control [14]. In particular, surface topography and the micro/nanostructures of biomaterials play an important role in cellular response. A biomaterial’s micro/nanostructure not only affects the form and migration of cells, but also influences the differentiation of stem cells [15,16,17,18]. Through different treatments, SF can be arranged to hold a broad range of forms, such as solution, powder, fibers, films, hydrogels, and sponges; this allows the use of SF for constructing many different scale structures ranging across the nano, micro, and macro [4,14]. In the past decades, the scientific community has investigated a great number of inherent structures of biological materials and their corresponding functions [19,20,21]. The structures of SF-based biomaterials are diverse; their ability to form particulate, fiber, film, and three-dimensional (3D) porous structures make them capable of unique biological functions. Many living organisms found in nature exhibit fascinating multiple functionalities arising from their multi-level surface structures. Inspired by the multifunctionality displayed by nature, the development of SF-based biomaterials is aimed to realize the possibility of fabricating biomimetic multi-level structures for multifunctional integration in various biological applications. In this review article, we summarize recent research progress in some typical single structure constructions of SF-based materials with different biological functions. Corresponding biomimetic SF-based materials with multi-level structures are also presented and discussed.

## 2. Physicochemical Properties of Silk Fibroin as Biomaterials

Silk fibroin was recognized by the US Food and Drug Administration (FDA) as a biomaterial in 1993 [4]. Compared with other natural biopolymers, SF is promising due to its excellent mechanical properties, good biocompatibility, biodegradability, and the versatility of structural re-adjustments. These advantageous properties are attributed to its unique physicochemical properties.

**Figure 1:**
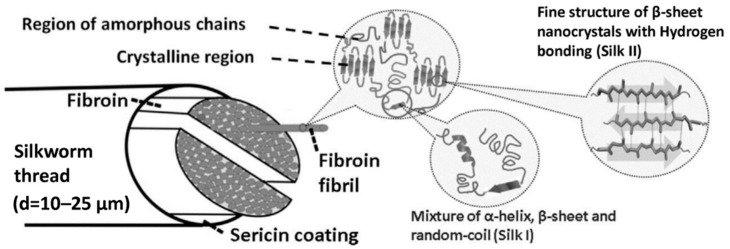
Schematic presentation of the silk fibroin (SF) structure; d represents the diameter of a single silkworm thread. Reproduced from [28].

Silk from silkworms is composed of two primary proteins: SF (approximately 75%) and sericin (approximately 25%) (Figure 1) [22]. In raw silk, sericin is positioned across the surface of two parallel fibroin fibers, binding them together. Silk fibroin is a fibrous protein with a semi-crystalline structure that provides stiffness and strength. Sericin is a glue-like amorphous protein that acts as an adhesive binder to keep the structural integrity of the fibers. Sericin is soluble and can be removed by a thermochemical process known as degumming [6,23]. Silk fibroin consists of a heavy (H) chain (~390 kDa) and a light (L) chain (~26 kDa) linked together via a single disulfide bond at the C-terminus of the H-chain, forming an H–L complex. A glycoprotein P25 (~25 kDa) is also non-covalently linked to the H–L complex. The H-chain, L-chain, and P25 are assembled in a ratio of 6:6:1 to form silkworm silk. The amino acid composition of SF from *Bombyx mori* consists mainly of Gly (43%), Ala (30%), and Ser (12%). The hydrophobic domains of the H-chain contain a repetitive hexapeptide sequence of Gly-Ala-Gly-Ala-Gly-Ser and repeats of Gly-Ala/Ser/Tyr dipeptides, which can form stable anti-parallel β-sheet crystallites. The amino acid sequence of the L-chain is non-repetitive, so the L-chain is more hydrophilic and relatively elastic [3,24,25,26,27]. The main crystal structures of silkworm SF are silk I and silk II. The little and unstable silk III structure also exists in regenerated SF solution at the air/water interface. Silk I is a metastable structure with crank or S zigzag structure spatial conformation, belonging to the orthorhombic system. Silk II is an anti-parallel β-sheet structure, belonging to the monoclinic system. Strong hydrogen bonds between adjacent segments contribute greatly to the rigidity and tensile strength of SF [3,4,28]. The silk I structure can be easily converted to silk II via methanol or potassium phosphate treatment [29,30,31].

## 3. Structure Design of Silk Fibroin-Based Biomaterials

**Figure 2:**
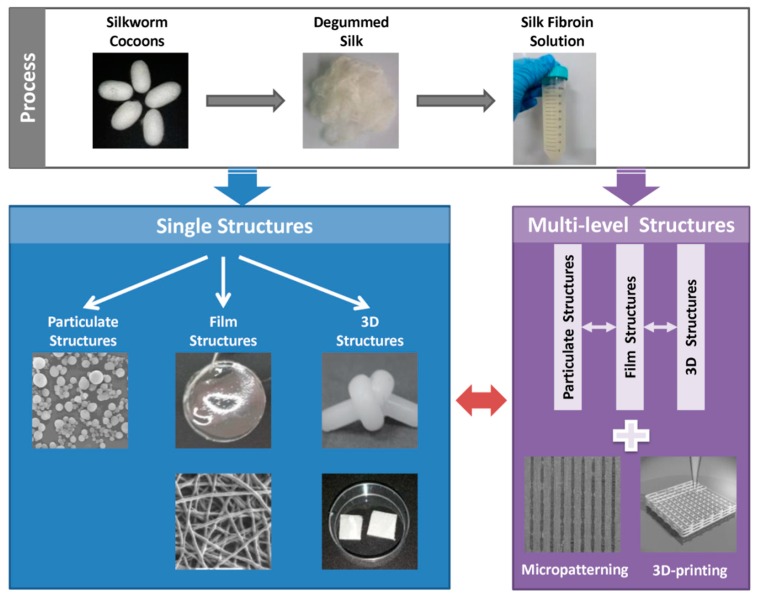
Structural design of SF-based biomaterials from single structures to multi-level structures. Reproduced from [34,35].

As SF possesses excellent processability, various forms of SF-based biomaterials can be fabricated using different treatments. To obtain the aqueous regenerated SF solution, silk is processed by the following steps (Figure 2). Degumming is the first step to processing raw silkworm silk. Then the degummed silk fibers are dissolved in a high molarity chaotropic salt solution such as LiBr or ionic liquids. The salts are subsequently removed via dialysis [32]. It is also possible to directly extract the SF from the posterior silk gland [33]. In the following section, we summarize the various structures of SF-based biomaterials, from single structures to multi-level structures, and discuss their corresponding biological functions.

### 3.1. Single Structures

#### 3.1.1. Particulate Structures

**Figure 3:**
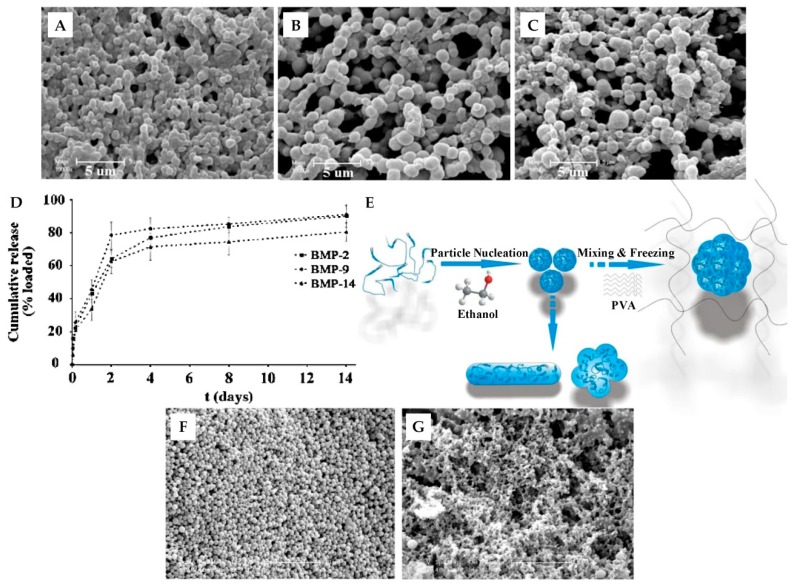
Scanning electron microscope (SEM) images (**A**–**C**) of SF microparticles fabricated with different SF: ethanol ratios, (**A**) 2:1, (**B**) 3:1 and (**C**) 4:1, scale bar: 5 µm; (**D**) Release kinetics of bone morphogenetic protein (BMP)-2, BMP-9, and BMP-14 immobilized in SF particles, with 0.5 µg of BMP per mg of SF. Reproduced from [38]; (**E**) Mechanism of SF particles’ regular formation (**F**) with addition of poly vinyl alcohol (PVA) and irregular formation (**G**) without addition of PVA, scale bar: 10 µm. Reproduced from [39].

Silk fibroin materials with particulate structures can be prepared using several methods. Self-assembly technology is widely used to prepare SF micro- and nanoparticles. The hydrophilic (Tyr, Ser) and hydrophobic (Gly, Ala) chain segments in SF molecules can be arranged alternately [36], allowing SF molecules to form micelles via a self-assembly mechanism. By adding a certain amount of ethanol and quenching below the freezing point, mild self-assembly of SF is initiated, producing 0.2 to 1.5 μm-sized SF microspheres without the involvement of toxic agents. The microsphere size is controlled by the concentration of SF, the freezing temperature and the amount of ethanol added (Figure 3A–C) [37,38]. Furthermore, Shi et al. reported that the addition of poly vinyl alcohol (PVA) improved the morphology of SF particles [39]. In the freezing process, PVA forms a hydrogel network to restrain the nucleation of SF (Figure 3E). Regular and smooth SF particles were formed under the influence of PVA (Figure 3F), unlike the unpredictable structures of SF particles produced without PVA (Figure 3G) [39]. In contrast, the phase separation of SF solution by salting out is relatively simple. Lammel et al. reported that SF particles obtained using potassium phosphate had controllable sizes ranging from 500 nm to 2 μm [40]. The secondary structure and zeta potential of the SF particles were affected by the pH value of the potassium phosphate solution [40]. Zeng et al. discovered that a lower and narrower molecular weight distribution of SF could promote the formation of SF microspheres with smoother surfaces and more uniform shapes [41]. In contrast to the above methods, milling SF is a physical method used to make SF particles. Milling does not use any chemicals, as SF is instead ground using machinery. The size of the resulting particles is influenced by the type of grinding miller and the aperture of the vibratory sieve shaker [42,43]. Various other methods for SF micro- and nanoparticle preparation have been reported, including desolvation [44], spray drying [45], laminar jet break-up [46], capillary microdot [47], and electrospray techniques [48].

Silk fibroin particles are primarily used in drug delivery applications. Wenk et al. found that SF spheres exhibited almost 100% encapsulation efficiencies for both salicylic acid and propranolol hydrochloride [46]. The encapsulation of insulin-like growth factor I (IGF-I) was also efficient. The IGF-I-loaded SF spheres were observed with a continuous release over seven weeks in bioactive form [46]. Moreover, small model drugs such as alcian blue, rhodamine B, and crystal violet were loaded to SF particles produced by salting out, based on simple electrostatic interactions. The in vitro release confirmed that the charge and the secondary structure of the SF particles affected the release of small drugs [40]. In addition, SF particles used as carriers for bone morphogenetic proteins (BMPs) provided a sustained release of BMPs over 14 days (Figure 3D), making them useful for bone tissue engineering [38]. SF nanoparticles have also been applied in wound healing. Lee et al. fabricated hydrocolloid dressings incorporating SF nanoparticles [49]. The experiments demonstrated that the adding of SF nanoparticles could improve structural stability of the dressing and increase cell growth rate. The SF nanoparticle hydrocolloid dressings (SFNHD) were also used in animal models for burn wound treatment. The results showed that SFNHD could reduce the burn size of rats and accelerate the growth of collagen fibers when compared to commercially available dressing, which indicated that SFNHD may be a better choice for wounds [49]. Bioimaging is a critical tool in drug delivery and therapeutics. Khalid et al. reported SF spheres encapsulating fluorescent nanodiamonds (NDs) by coflow technique for long-term biotracking and imaging. The SF encapsulated NDs were used to research intracellular mobility in vitro, which showed enhanced mobility, increased diffusion, and higher fluorescent brightness compared to bare NDs [50].

#### 3.1.2. Film Structures

**Figure 4:**
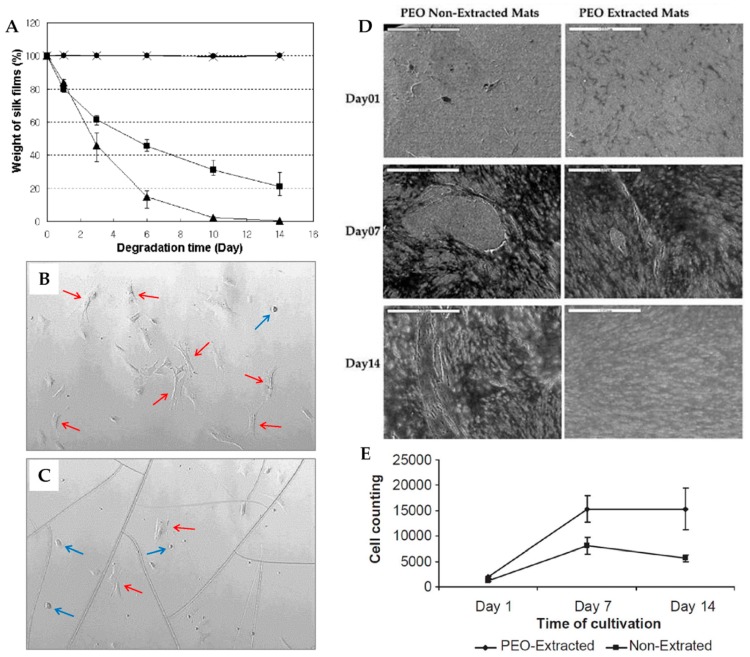
(**A**) Enzymatic degradation of SF films. (▲: SF films by water annealing in enzyme solution; ■: SF films by methanol treatment in enzyme solution; ×: SF films by water annealing in PBS; ●: SF films by methanol treatment in PBS), *n* = 5; Human bone-marrow stromal cells (hMSCs) attachment at 2 h on water-annealed SF film (**B**) and methanol-treated SF film (**C**). The cracks on the film in image (**C**) are induced by methanol treatment. **Red** arrows indicate fully attached cells and **blue** arrows indicate attaching cells. Reproduced from [56]; (**D**) SEM images of bone-marrow stromal cells (BMSCs) growing on polyethylene oxide (PEO) non-extracted and PEO extracted SF mats, respectively, after 1, 7, and 14 days. Scale bar: 500 µm; (**E**) Proliferation of BMSCs grown on SF mats. Seeding density: 2.5 E4 cells/cm^2^, *n* = 4. Reproduced from [59].

Silk fibroin materials with film structures usually take the form of films and mats. Silk fibroin films are typically prepared by casting the aqueous fibroin solution [51]. Other reported techniques include spin-coating [52], vertical deposition [53], and spin assisted layer-by-layer assembly [54]. Silk fibroin films can be easily obtained by casting SF solution on a smooth and clean plate with subsequent natural evaporation or drying under a certain temperature. Sagnella et al. reported a vertical deposition method to produce films. In their report, a glass coverslip was inserted vertically in the SF solution in an oven at 50 °C; and because of the lateral capillary force and surface tension drive between the fibroin solution and the glass coverslip, silk fibroin solution could be deposited on the glass coverslip and dried to obtain the transparent SF film. However, films prepared by this method showed a non-homogeneous texture, which was caused by the inherent feature of the vertical deposition method [53]. Jiang et al. developed an ultrathin SF film (45 ± 5 nm) fabricated by the layer-by-layer assembly technique; the resulting film displayed high elastic modulus and ultimate tensile strength due to its gradual self-reinforcing structure [54]. The stability of SF films used as biomaterial is significant; this property can be improved by stretching [55], water annealing [56], slow-drying [57], and alcohol immersion [58]. Methanol or ethanol are most often used to prepare water-insoluble SF films with increased β-sheet content. Minoura et al. reported that SF films treated with methanol showed high oxygen and water vapor permeability, as well as good mechanical properties [51]. Terada et al. discovered that ethanol concentration could influence the surface properties of the films. When less than 80% ethanol was used to treat the SF film, the outermost layer of fibroin film looked like jelly, while after treatment with great than 90% ethanol, the film surface was harder, which had an impact on the adhesion and aggregation of cells [52]. However, alcohol-treated films are extremely brittle and less transparent, which restricts practical uses [51,56]. Demura et al. presented a glucose oxidase (GOD)-immobilized SF membrane, which was physically stretched in an apparatus with Clark-type oxygen electrode to induce structural transition from the random coil (silk I) to β-sheet (silk II) without chemical treatments [55]. Silk fibroin films treated with the above techniques exhibited slow biodegradation due to their high β-sheet content. To emphasize the need of SF biomaterials with increased degradation rates (Figure 4A), water-stable fibroin films with reduced β-sheet content were prepared by water annealing. Jin et al. found that after being annealed in water for 24 h, the SF films formed stable Silk I structure and did not transform to Silk II with methanol or stretching treatment. Additionally, the water-annealed SF films were more transparent and could avoid cracks induced by methanol treatment (Figure 4B,C) [56]. Similarly, Lu et al. developed water-insoluble SF films with a silk I structure by very slow drying; the resulting films had a faster enzymatic degradation rate and better mechanical ductility [57].

Silks are fibrously structured in nature. For the purpose of mimicking the structure and biological function of extracellular matrix (ECM), fibers are incorporated into two-dimensional (2D) mats, which have large surface areas and porous structures [14]. Electrospinning is the most commonly used technique to prepare SF mats due to its flexibility and versatility. The morphology and secondary structure of SF mats can be adjusted by tuning the electrospinning voltage, SF concentration, flow rate, and receive distance [60,61]. At low SF concentrations, clustered or beaded fibers may occur in the collector. The best electrospinning conditions have been widely investigated to obtain optimal SF mats [61,62,63]. In general, SF has been electrospun with spinning solvents such as polyethylene oxide (PEO), hexafluoroisopropanol (HFIP), hexafluoroacetone (HFA), and formic acid, which can decrease biocompatibility. Jin et al. reported that when SF/PEO electrospun mats were washed with water for two days in order to remove PEO, the original morphology and structure of the SF mats did not change [59]. Chen et al. reported a method to obtain the SF mats by an electrospinning process using a highly concentrated, all-aqueous SF solution. The electrospun SF mats exhibited belt-like fibers, with a breaking stress and strain of 1.49 MPa and 1.63%, respectively [64]. The electrospinning method can be used to easily prepare composite SF mats with unique functions. Cellulose nanowhiskers (CNWs) were added to the SF solution to reinforce the mats with twice the tensile strength and Young’s modulus [65]. Additionally, the doping of Ag [66] and TiO_2_ [67] nanoparticles was found to improve the antibacterial property of SF mats.

Silk fibroin biomaterials with film structures are widely used for artificial skin, wound dressing and drug delivery. Bone marrow-derived mesenchymal stem cells (BMSCs) seeded on the SF films showed better cell proliferation and lower inflammatory reaction in vivo, compared to cells seeded on tissue culture polystyrene (TCPS) or collagen [68]. Human bone-marrow mesenchymal stem cells (hMSCs) preferred to attach fully on water-annealed SF films rather than methanol-treated films (Figure 4A,B) [56]. Silk fibroin/chitosan films exhibited advantageous mechanical properties and good water vapor and oxygen permeability, making them comparable to commercial wound dressings [69]. Furthermore, SF mats coated with silver nanoparticles (AgNPs) were revealed to have effective antibacterial activity with a relatively low concentration of ionic silver compared with commercial wound dressing. Therefore, SF has potential commercial application as antimicrobial wound dressings [66]. Jin et al. observed that BMSCs grew to a higher density on PEO extracted SF mats compared to PEO non-extracted mats, as confirmed by scanning electron micrographs (Figure 4D) and cell counting (Figure 4E) [59]. Hybrid SF with vitamin E provided enhanced inoxidizability of mouse skin fibroblasts, suggesting promising applications in skin care [70]. It was found that the structure and properties of SF films affected the release rate of drug delivery. SF films exhibited a slower doxorubicin release with smaller nanostructure and more β-sheet structure content [58]. Ceftazidime (CTZ) was successfully encapsulated into SF/gelatin mats and showed antibacterial effects during a release process of over six hours [71]. Recently, film structured SF biomaterials have also been used in guiding bone regeneration. Cai et al. developed a novel SF membrane via lyophilization, densification, and ethanol treatment. The osteoconductive potency of the SF membrane was investigated in a rabbit calvarial defect model. The results showed that the SF membrane could prevent connective tissue invasion into the defected area and had a similar amount of new bone and defect closure compared to collagen membrane [72]. Jia et al. found that poly-D-lysine(PDL)-optimized SF films could promote corneal epithelial cell proliferation and viability. The PDL-optimized SF films served as substrates for human corneal epithelium formation, which paved a new path for corneal tissue regeneration [73].

#### 3.1.3. Three-Dimensional Structures

**Figure 5:**
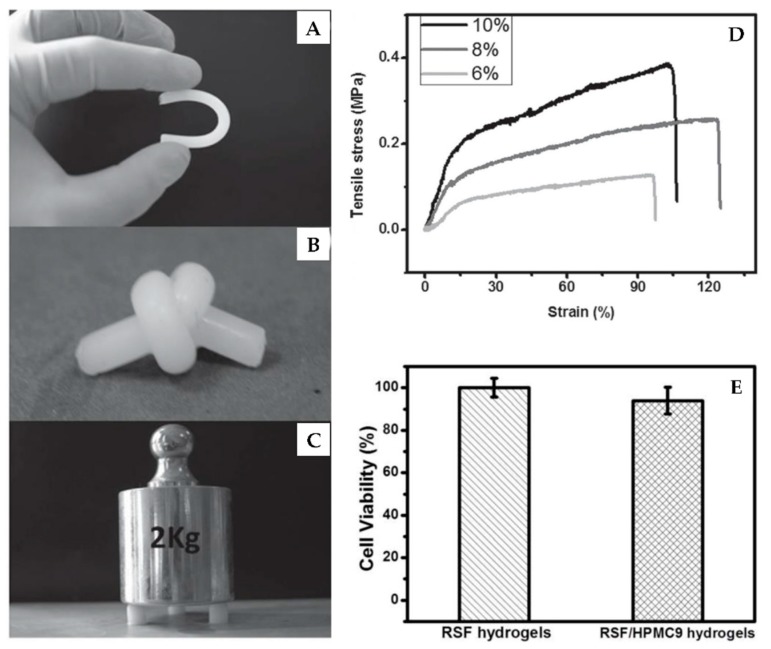
Images of regenerated silk fibroin (RSF)/hydroxypropyl methyl cellulose 9 (HPMC9) hydrogels’ reaction to bending (**A**), knotting (**B**) and compressing (**C**); (**D**) Representative tensile curves of RSF/HPMC9 hydrogels with different solid contents; (**E**) Cytotoxicity test of mouse fibroblast cells cultivated with RSF hydrogels and RSF/HPMC9 hydrogels; RSF/HPMCP9: the ratio of RSF to HPMC was 9/1. Reproduced from [34].

Three-dimensional structures of SF usually exist as hydrogels and sponge materials. Hydrogels possess an interconnected network structure with high water content. The gelation of SF can be induced by sonication [74], vortex [75], heating [76], solvent treatment [77], photo-crosslinking [78], and electrogelation [79]. The rate of the gelation process is controlled by temperature, pH, fibroin concentration, and addition of other compounds. Faster SF gelation is produced by higher concentration, lower pH values, higher temperature, and the addition of Ca^2+^. During the sol–gel transition process of SF, secondary structural changes occur from a random coil state to a β-sheet conformation [80,81]. However, Lu et al. reported that the electrogelation of SF resulted in transformation from random coil to α-helix instead of β-sheet. The formation of this intermediate structure is vital in electrogelation [79]. Ultrasonication is a novel method that produces SF hydrogels with significantly increased gelation rates, resulting in a change to the hydrophobic hydration and formation of SF with stable β-sheet structures. In fact, ultrasonication can cause temperature increase, mechanical and shear forces, and increased air–liquid interfaces, which accelerate the gelation process [74]. Kim et al. discovered that regulation of the alkaline hydrolysis time allowed production of SF solutions with different molecular weights. Due to longer hydrolysis destroying hydrophobic segments, SF with smaller molecular weights caused gel time to increase. The molecular weight of SF also influenced the microstructure and physical properties of hydrogels. Silk fibroin with shorter chains produced hydrogels with smaller structural units and higher porous network structures. Additionally, physical properties such as shear elastic modulus varied under different molecular weights of SF. In general, due to their relatively loose network structures, the shear elastic modulus of SF hydrogels decreased with use of smaller molecular weights [82]. SF/nanohydroxyapatite (nanoHA) composite hydrogel was prepared by adding ethanol as a gelling agent; this hydrogel exhibited an interconnected porous structure. The nanoHA particles were uniformly distributed in the composite hydrogel, and the compression modulus was found to increase with increasing nanoHA concentration [77]. Luo et al. developed a SF/hydroxypropyl methyl cellulose (HPMC) hydrogel with remarkable mechanical performance, fabricated by simple mixing and heating (Figure 5). Both the compressive modulus and tensile modulus of the hydrogel were over 1 MPa, and the break energy was as high as 3500 Jm^−2^. These advantageous properties were attributed to the dominant crosslinks of smaller β-sheet structures in the SF/HPMC hydrogels [34]. Partlow et al. have reported a new method to fabricate highly tunable elastic SF hydrogels via enzymatically covalent crosslinking of tyrosine residues in SF generated by horseradish peroxidase (HRP) and hydrogen peroxide. The new hydrogels could bear shear strains approximatively 100%, compressive strains greater than 70%, and show stiffness between 200 and 10,000 Pa, which included numerous properties of native soft tissues. The HRP SF hydrogels also exhibited controllable kinetics and could maintain high resilience and resistance to fatigue under different molecular weights and solvent compositions [83]. Yan et al. developed core-shell SF hydrogels with spatially controlled conformation by immersing the enzymatically crosslinked SF hydrogels in methanol for 0–10 min. The shell layer presented compact morphology with dominant β-sheet conformation and the core layer exhibited porous structure with mainly random coil conformation confirmed by SEM and Fourier transform infrared spectroscopy (FTIR) [84].

**Figure 6:**
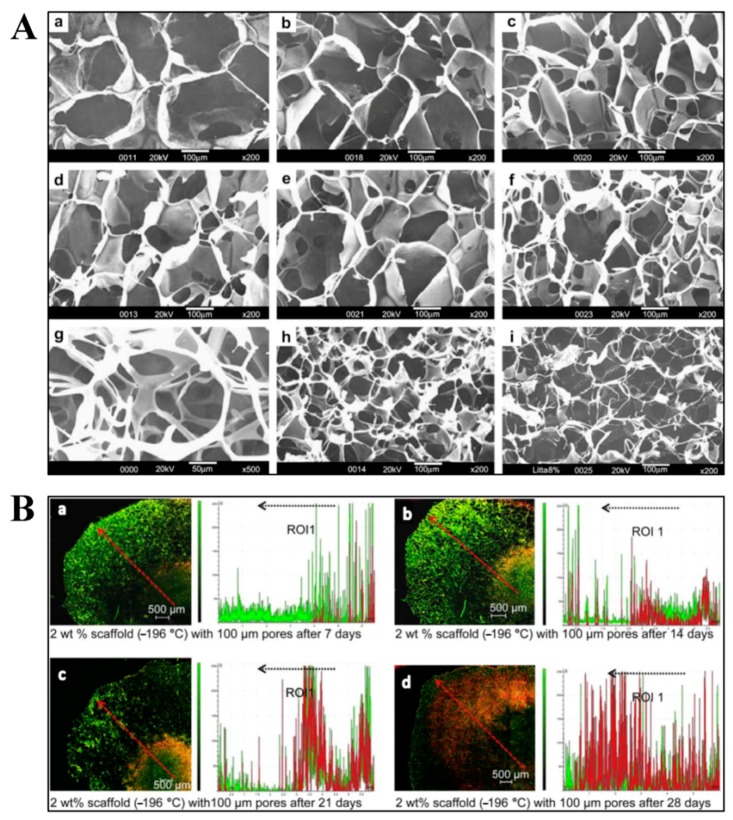
(**A**) Scanning electron microscope images of SF scaffolds fabricated by freeze-drying technique using (**a**–**c**) 2 wt % SF at −20 °C; (**d**–**f**) 4 wt % SF at −80 °C; and (**g**–**i**) 6 wt % SF at −196 °C; scale bar: (**a**–**f**,**h**,**i**): 100 µm; (**g**): 50 µm. (**B**) Confocal laser micrographs of human dermal fibroblast cell migration on SF porous scaffolds fabricated at −196 °C at different time points. The cells are stained with Hoechst 33342 for nuclei (green) and Rhodamine–phalloidin for actin filaments (red); scale bar: 500 µm. The black dotted arrows indicated the region and direction corresponding to the red dotted arrows on the previous graphs; ROI: region of interest. Reproduced from [88].

Three-dimensional SF sponges possess interconnected porous structures, and can be obtained by salt leaching, gas foaming, and freeze-drying [85]. For the salt leaching technique, NaCl particles are usually used as the porogens. Salts are added into SF solution in a container, and then extracted from the sponges by immersion in water [85,86,87]. Kim et al. reported that the pore sizes and porosity of sponges can be controlled by regulating NaCl particle size and SF solution concentration. At higher SF concentrations and larger NaCl particle sizes, homogeneous matrices with uniform pore size distributions were formed [87]. Hexafluoroisopropanol and methanol are always used in the treatment processes of SF porous sponges. An all-aqueous processing technique was used to fabricate porous SF scaffolds without the addition of organic solvents. The results illustrated that the aqueous-derived SF sponges exhibited a more uniform and highly interconnected morphology, as well as a faster degradation rate in the presence of protease, compared with HFIP-derived sponges [87]. On the other hand, the pore sizes of the lyophilized SF sponges can be controlled by freezing temperature and fibroin concentration. Mandal et al. reported that pore sizes ranging from 200–250 µm were obtained by freeze-drying at −20 °C, while smaller pore sizes ranging from 100–150 µm, and 80–100 µm were observed at −80 and −196 °C, respectively (Figure 6A). At a fixed freezing temperature, pore size decreased with increasing SF concentration. Sponges with a high porosity of 96% were fabricated by freeze-drying at −196 °C with 2 wt % SF [88]. SF sponges can also be prepared using the gas foaming technique, which is conducted by adding ammonium bicarbonate into fibroin solution. NH_4_HCO_3_ particles are then sublimated in hot water, thereby inducing the porous sponge structure [85]. Tamada et al. reported a novel method called freeze-thaw, which formed SF porous sponges via the addition of organic solvents such as methanol, ethanol, and propanol. The mixed solution was frozen and then immersed in buffer solution or water to remove the solvents. Silk fibroin sponges made by this method showed good tensile strength and compressive modulus due to the existence of silk II crystalline structures induced by organic solvents [89]. Yan et al. combined salt-leaching and freeze-drying methodologies to prepare SF scaffolds with high-concentration aqueous solutions. Sodium chloride particles (500–1000 μm) were used as porogens added into SF solution and then extracted by distilled water. Next, the scaffolds were frozen at −80 °C for one day and then freeze-dried. The prepared SF scaffolds possessed high porosity and interconnectivity with homogeneous macro/microporous structures. Importantly, after in vitro degradation for 30 days, the SF scaffolds could maintain their original structure and morphology integrity, as well as their mechanical properties. Therefore, the SF scaffolds showed potential use in meniscus and cartilage regeneration [90].

Silk fibroin as three-dimensional structures are ideal material for tissue engineering because 3D structure biomaterials mimic the in vivo physiological environment more closely than 2D structures [91]. Silk fibroin hydrogels can be used in cell encapsulation. Wang et al. reported that hMSCs can be successfully encapsulated in sonication-induced hydrogels, which can keep proliferation for weeks, and maintain cell activity and function for more than 21 days [74]. Yucel et al. found that vortex-induced SF hydrogels showed a shear-thinning behavior when injected through needles. However, the stiffness of the hydrogels recovered rapidly after injection, which could provide the basis for injectable cell delivery scaffolds [75]. Yan et al. found that the HRP crosslinked SF hydrogels could spontaneously undergo conformation changes from random coil to β-sheet. The SF hydrogels could support ATDC-5 cells survival up to seven days; however the subsequent β-sheet transition resulted in cell apoptosis. Furthermore, HeLa cells were incorporated in the hydrogels to research the in vivo chick chorioallantoic membrane model for tumor formation. The results showed that angiogenesis and tumor formation were suppressed by SF hydrogels due to the conformational changes. These SF hydrogels could be used as a biomimetic platform to modulate encapsulated cell fate and suppress cancer formation [92]. Interconnected porous SF sponges are able to support cell attachment, proliferation, and differentiation due to their convenient transport of nutrient and waste. In highly interconnected SF scaffolds, human dermal fibroblast cells migrated within the interconnected pores, and had been observed to reach scaffold periphery after culturing for 28 days (Figure 6B) [88]. Silk fibroin hydrogels incorporated with nanoHA showed enhanced metabolic activity and alkaline phosphatase activity of the osteoblastic cells, which could be applied in bone tissue engineering [77]. Mouse fibroblast cells were used to test the cytotoxicity of SF/HPMC hydrogels, and the results showed that the cell survival rate was over 95% (Figure 5E). Therefore, it had been demonstrated that the addition of HPMC observably improved mechanical properties but had little influence on the biocompatibility of SF. Thus, the applicability of SF could be expanded for load-bearing biomaterials [34]. SF scaffolds were also reported in spinal cord injury repair. Zhang et al. fabricated a 3D multichannel/laminin SF scaffold with oriented ridges by a novel directional freeze-drying technique. The multichannel SF scaffolds were implanted in Sprague-Dawley rat spinal cords for bioactivity evaluation. It was found that the SF scaffolds could mediate cell migration, promote blood capillary formation, and help axonal extension, which suggested the application of the multichannel SF scaffolds for spinal cord injury regeneration [93]. Han et al. prepared water-insoluble SF scaffolds containing physical cues instead of growth factors for vascularization. The SF-based scaffolds could improve cell differentiation into endothelial cells and promote neovascularization, and eliminate many inherent disadvantages caused by growth factors at the same time [94].

### 3.2. Multi-Level Structures

#### 3.2.1. Micropatterning Structures

As early as in 1912, Harrison, the pioneer of contact guidance phenomenon, proved that cells can grow along the direction of spider silk. Harrison first reported the influence of topological structure on cell behavior, which promoted research in the field of protein and cell micropatterning [95]. The organization of the ECM is complex and hierarchical with micro- and nanotopography, which have a vital role in affecting cell behavior. Cells can respond to the surrounding environment and show different behaviors. Micro- and nanostructures of biomaterials have shown great importance in guiding cell migration, as well as in influencing cell adhesion, proliferation, and differentiation [96,97].

**Figure 7:**
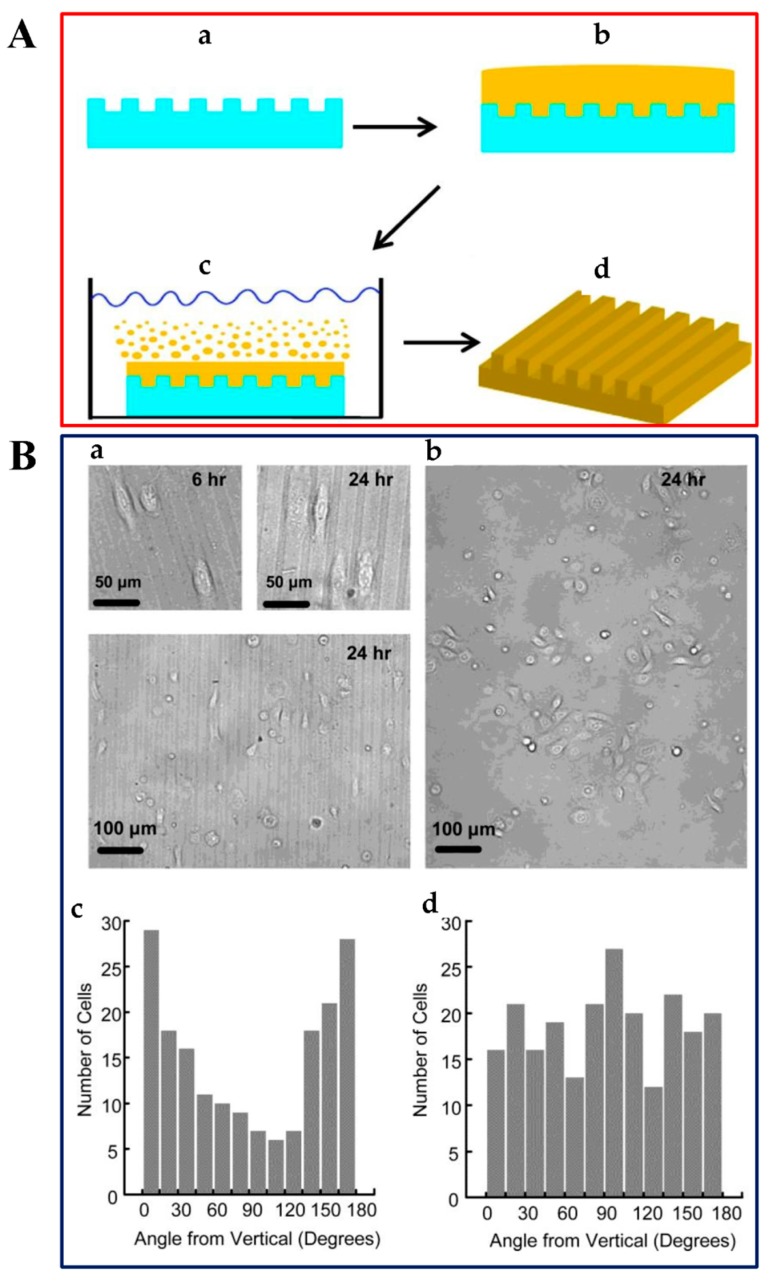
(**A**) Schematic illustration of the production of patterned SF films: (**a**) Pre-designed polydimethylsiloxane (PDMS) stamp; (**b**) Spin-coating SF solution on the PDMS; (**c**) Extracting the ionic liquid solvent in a methanol bath; (**d**) Peeling the crystallized patterned SF film from the stamp; (**B**) Data of cell alignment on patterned SF films as compared to the unpatterned (collagen-coated) films: Optical micrographs of keratinocytes growing on (**a**) patterned SF films at 6 and 24 h and (**b**) unpatterned film at 24 h; Histograms of cell alignment on (**c**) patterned SF films and (**d**) unpatterned films. The *x*-axis represents cell angle; the *y*-axis represents cell counts. Adapted with permission from [101]. Copyright (2007) American Chemical Society.

Photolithography is a widely used and traditional method for fabricating micropatterning SF biomaterials. The photolithography technique is based on a photomask with micro/nanoscale patterns and photoresist. The photoresist is spin-coated on a substrate and then the photomask covers on the photoresist. Because of the photo-sensitive property of photoresist, regions exposed to certain light source through the photomask will be decomposed. In this manner, the patterns of the mask are transferred to the substrate [98]. Park et al. reported the use of pure SF as a positive-tone photoresist in lithography; this process did not require photoinitiators, and water was the only chemical used. Silk fibroin solution was first spin-coated onto the silica substrate, then SF film was illuminated by ArF excimer laser through a patterned Cr mask. After developing the exposed area with distilled water, the patterned SF film showed diffracted colors with a minimum line width of 1 µm [99]. Another technique is soft lithography, which is based on molding and printing with an elastomeric stamp to realize pattern transfer from the template. Compared to traditional photolithography technique, soft lithography is highly effective, convenient, and inexpensive [100]. Polydimethylsiloxane (PDMS) is commonly used as the elastomeric stamp mold. Gupta et al. reported micropatterning SF films by soft lithography. First, SF dissolved in an ionic liquid was casted onto a PDMS stamp with microchannel, and then immersed in methanol to crystallize the SF film and extract the ionic liquid solvent. Finally, the patterned SF film was peeled from the substrate with a peak-to-peak periodicity of 6.6 µm (Figure 7A) [101]. Optical-grade SF film with 3D diffraction nano- and micropatterns were also fabricated using soft lithography [102]. Zhu et al. presented a template-assisted electrospray deposition (ESD) technique to create precise designed micropatterning SF/nanoHA composites on the Ti substrate. Silk fibroin and nanoHA composite was forced from the needle by the syringe pump, and then sprayed onto the template-covered substrate under an applied electric field. The SF/nanoHA micropatterns showed uniform topography, and the morphological properties of the nanocrystals were not influenced by the ESD technique [103]. The electron beam lithography technique uses a focused electron beam to expose the resist on the substrate, which can produce nanometer scale patterns under the control of computer. The fabrication of linewidths as small as 5–7 nm using 100 keV electron beam lithography, has been reported [104]. Du et al. used polymethylmethacrylate (PMMA) as resist on silicon wafer, subsequently preparing the patterned silicon substrate by electron beam lithography. Following this process, SF solution was poured onto the patterned substrate and dried at room temperature. Methanol treatment was incorporated to render the films water insoluble. Various substrate topographies of SF films, such as square pillars, square wells, and gratings, were obtained via this method [96]. Polystyrene (PS) colloidal crystal template technique is used to organize ordered interconnected SF inverse opals. By capillary infiltration, SF solution is intercalated into the predefined template. After treating the dried samples with ethanol, the polystyrene template is removed by toluene [105]. You et al. reported SF microsphere array patterns made through a polystyrene microsphere self-assembly template. First, PS microspheres were dropped onto a glass substrate to form a monolayer array. Next, PDMS was cast onto the previous substrate, and then peeled off after solidification. Finally, SF solution was poured onto the PDMS mold to obtain micropatterned SF films with sphere diameters of 8 µm [106]. Additionally, scanning probe lithography (SPL) is a high resolution patterning technique that uses a tip to image features on a substrate. Atomic force microscopy (AFM), a type of SPL, has been reported to directly deposit the relatively hydrophobic SF onto mica under liquid. The AFM tip produced SF micropatterns on mica in both contact and tapping modes [107].

Filopodia of cells act as antennae to detect the microenvironment and send messages to nucleus. Conversion of filopodia to lamellipodia is the main driving force of the directional extension of lamellipodia, which is a prerequisite for the spreading and migration of BMSCs. Accelerating lamellipodia formation, which does not occur on flat substrates, was observed on the microsphere patterned SF surface. The spreading of BMSCs was guided along the microsphere arrays by directional lamellipodia extension. Filopodia also served as a skeleton, wrapping around the microsphere to guide cell migration. Therefore, patterned SF films can promote cell adhesion and proliferation due to faster lamellipodia formation and cell spreading [106]. Gupta et al. reported that SF films with periodic grooves (spacing of 10 µm, depth of 6 µm) can affect cell behavior. Keratinocytes grown on the micropatterned SF films revealed preferential alignment along the groove pattern even after culturing for 24 h. Cell angles, defined as the angle between the vertical direction of the optical graph and the long axis of a cell, indicated that the cells prefer to elongate and orient along the direction of the silk film patterns, as demonstrated by the large number of cells with a cell angle of 0° and 180°. However, cells grown on the unpatterned films showed no preferential orientation behaviors, which could be seen in the optical micrographs and the histograms of cell alignment (Figure 7B) [101]. Human umbilical vein endothelial cells (HUVECs) were used to investigate cell behaviors on different patterned SF thin films. The results showed that some filopodia of cells could align perpendicular to the grooves but not to the ridges, and some filopodia could cross over the round wells or grow along the borders on the round wells and square pillars substrate. HUVECs orientation and alignment were observed on grating patterns with greater than 800 nm pitch and 400 nm depth. These studies are useful in realizing the importance of incorporating micro- and nanostructures in biomaterials [96].

**Figure 8:**
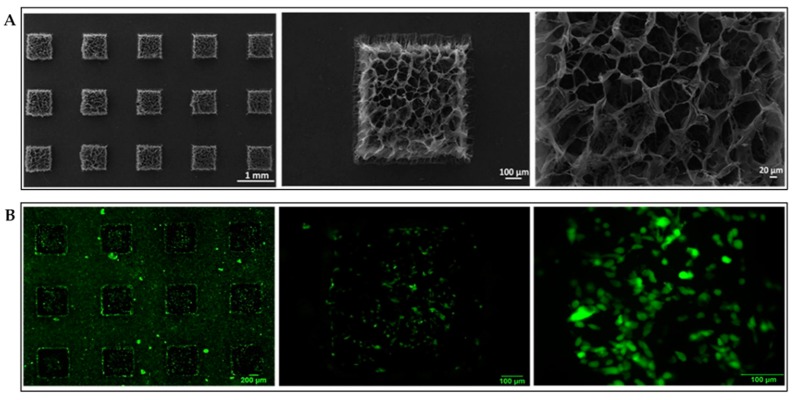
(**A**) Scanning electron microscope images of micropatterned SF/gelatin methacrylate (GelMA) porous scaffolds; (**B**) Fluorescence images (2×, 10×, and 20×) of NIH-3T3 fibroblast cells stained with live/dead viability kit after cultured on the micropatterning scaffolds for one day. Reproduced from [108].

Most micropatterning SF biomaterials have flat surfaces in the microregions. To obtain the multi-level structures, the micropatterning technique should be combined with various micro- and nanostructures. Xiao et al. reported SF/gelatin methacrylate (GelMA) micropatterning porous scaffolds, which were produced by photolithography and lyophilization techniques. The micropatterning SF/GelMA scaffolds showed interconnected and open porous structures (Figure 8A), which were beneficial for diffusion of nutrients, oxygen, and metabolites, and provided a 3D support for cell growth. NIH-3T3 fibroblast cells cultured on the micropatterning SF/GelMA scaffolds distributed uniformly across the surface and exhibited excellent viability for one day (Figure 8B). The 3D microscaffold has potential use as traditional cell-laden microgel for bottom-up assembly to make biomimetic tissue constructs. The micropatterned porous SF scaffolds are expected to be used for various types of cells growth that are harvested to assemble tissue grafts in the future [108].

#### 3.2.2. Three-Dimensional Printing Structures

Scaffolds with multi-level pore size distribution exhibit multiple functions compared to traditional 3D porous structures. After the Ca^2+^ induced SF multiphase freeze drying, multipore sized SF scaffolds were obtained. It was demonstrated that the tiny pores on big pore walls help cell proliferation outwards and allow more cells to grow in the scaffolds. The 3D SF scaffolds with multi-level porous structures showed enhanced biological performance and maintained the mechanical performance at the same time [109].

**Figure 9:**
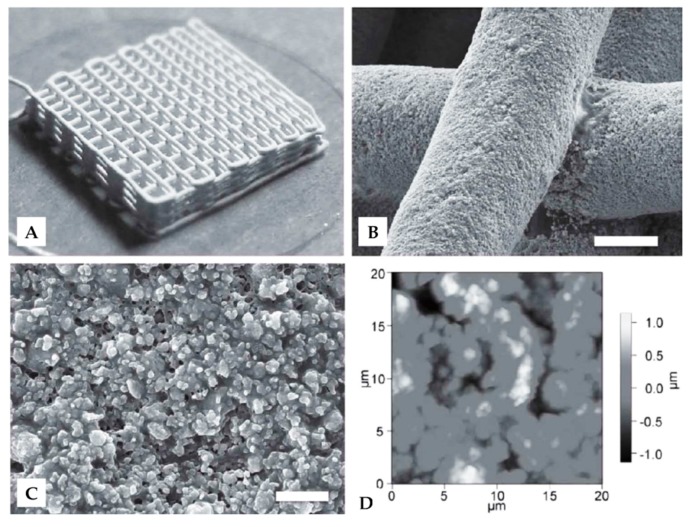
(**A**) Optical image of three-dimensional printing (3DP) silk/hydroxyapatite (HA) scaffold; (**B**) Scanning electron microscope image of individual silk/HA filaments at intersection. Scale bar: 100 µm; (**C**) Higher magnification image of the silk/HA filament surface. Scale bar: 10 µm; (**D**) Height profile of a representative silk/HA filament observed by atomic force microscopy (AFM). Reproduced from [113].

Three-dimensional printing (3DP) is a rapid prototyping (RP) technology that utilizes computer-aided design (CAD) model for layer-by-layer fabrication of 3D objects. Traditional methods of fabricating biomaterials fail to control their structures and internal geometry. The use of 3DP allows accurate, computer-controlled repetition of desired internal architectures and structures [110,111]. Three-dimensional printing has been used as an emerging technology in engineering, manufacturing, art, and many other areas. In recent years, 3DP has been applied in the field of biomaterials to meet the need for organs and tissues. However, 3D bioprinting is more complicated due to limited materials, choice of cell types, and technical challenges, among others [112]. Ghosh et al. used concentrated SF solution (28–30 wt %) as ink in the 3D direct writing of microperiodic scaffolds. The ink was deposited through a fine nozzle to form a precisely controlled complex array with 5 µm diameter silk fibers, in a layer-by-layer sequence. Different SF 3D structures were obtained, such as square lattice and circular web. Compared to standard pellet culture, the printed 3D scaffolds were observed to support HMSC adhesion and growth, and also enhance the chondrogenic differentiation of the cells due to the increased production of glycosaminoglycan [35]. Three-dimensional silk/hydroxyapatite (HA) scaffolds with gradient pore spacings ranging from 200 µm to 750 µm were developed by direct-write printing using SF ink containing HA particles. The multi-level scaffolds were printed in the form of a 3D lattice composed of interconnected silk/HA filaments (Figure 9A). Individual silk/HA filaments with little deformation of underlying layers were observed by SEM (Figure 9B). In an SEM image with higher magnification, HA particles were present in single filaments and SF acted as a binder to bind the HA particles together and promoted binding at each filament intersection (Figure 9C). The surface roughness of the multi-level SF/HA scaffolds was 467 ± 61 nm, as determined by AFM (Figure 9D). The silk/HA scaffolds with gradient pore sizes could support the co-cultures of hMSCs and human mammary microvascular endothelial cells (hMMECs). 3D direct writing technique has advantages in producing optimal silk/HA scaffold features for the formation of both bone tissue and vascular tissue in a single construct system [113]. Suntivich et al. presented an inkjet printing process using SF to fabricate nests for cell hosting. The printed SF nests were circular arrays with diameters of 70–100 µm, and were modified with ionic pairing to form silk II secondary structure. These ‘locked-in’ SF nests can be printed on any type of substrate to provide a platform for the incubation and proliferation of *Escherichia coli* cells [114]. Three-dimensional bioprinting SF-gelation scaffolds were applied in the culturing of human nasal inferior turbinate tissue-derived mesenchymal progenitor cells. Gelation of SF was processed by physical crosslinking (sonication) and enzymatic crosslinking (mushroom tyrosinase). The results showed that enhanced osteogenic differentiation was only observed on sonicated scaffolds due to the higher β-sheet content [115]. Recently, Rodriguez et al. reported a SF-based bioink for 3DP. Gelatin and glycerol were added in SF as bulking agent and physical crosslinking agent, respectively. The SF-based bioink was successfully printed into a specific cheek geometry according to the computed tomography (CT) scans from a patient with head and neck tumors. Moreover, the in vivo test of the 3DP SF implants showed that the material could retain shape up to three months with minimal inflammatory response and promote tissue integration in a mouse model [116]. Due to its unparalleled advantages, 3D printing will play an important role in future fabrication of biomaterials. With the aid of CAD modelling, virtually any structure can be printed. Currently, SF 3D printing focuses on single structure construction and rapid modeling of SF. However, as 3DP techniques mature, more attention will be directed to the construction of multi-level structures with excellent multifunctions.

## 4. Conclusions and Outlook

Silk fibroin, as a natural biological polymer, has turned out to be a kind of amazing biomaterial due to its tunable degradation, unique biomedical and mechanical performance, ease of processing, and sufficient supply. In summary, we have discussed the various structure constructions of SF under different processing techniques. Single structures of SF materials include particles, film structures (films and mats), and 3D structures (hydrogels and sponges). Although single structured SF-based biomaterials inspired by natural materials have been constructed successfully in the past few decades, more recent developments have turned to fabrication of multi-level structural SF-based materials. New techniques such as micropatterning and 3D printing, in combination with single structures, are used for fabricating SF multi-level structures. Cell adhesion, migration, proliferation, and differentiation are strongly affected by the different scale structures of SF biomaterials. From single structures to multi-level structures, SF biomaterials must exhibit multifunction integration, and a wide range of biological applications. With the development of micropatterning and 3D printing techniques, SF can be precisely constructed in predesigned specific structures to meet various tissue requirements. Recent advancements in understanding SF structures and processing open up new opportunities in the use of various forms of SF in biological applications. However, there are currently only a few studies on fabricating SF multi-level structures using micropatterning and 3D printing techniques. Therefore, the development of viable fabrication methods for constructing multi-level SF material structures will become the focus of future research in this field. The studies conducted in this increasingly popular area of research will be useful for understanding structure-multifunction relations and exploring multi-level structure design laws. We firmly believe that SF-based biomaterials with biomimetic multi-level structure have a bright and promising future.
